# Virulence Profile, Antibiotic Resistance, and Phylogenetic Relationships among *Escherichia coli* Strains Isolated from the Feces and Urine of Hospitalized Patients

**DOI:** 10.3390/pathogens11121528

**Published:** 2022-12-13

**Authors:** José F. Santos-Neto, Ana C. M. Santos, Júllia A. S. Nascimento, Liana O. Trovão, Fernanda F. Santos, Tiago B. Valiatti, Ana C. Gales, Ana L. V. R. Marques, Isabel C. Pinaffi, Mônica A. M. Vieira, Rosa M. Silva, Ivan N. Falsetti, Tânia A. T. Gomes

**Affiliations:** 1 Laboratório Experimental de Patogenicidade de Enterobactérias, Disciplina de Microbiologia, Departamento de Microbiologia, Imunologia e Parasitologia, Escola Paulista de Medicina, Universidade Federal de São Paulo, São Paulo 04023-062, Brazil; 2 Laboratório Alerta, Disciplina de Infectologia, Departamento de Medicina, Escola Paulista de Medicina, Universidade Federal de São Paulo, São Paulo 04039-032, Brazil; 3 Irmandade da Santa Casa de Misericórdia de Mogi Guaçu, Mogi Guaçu 13840-005, Brazil; 4 Laboratório Santa Cruz Medicina Diagnóstica, Mogi Guaçu 13840-052, Brazil; 5 Laboratório de Enterobacterias, Disciplina de Microbiologia, Departamento de Microbiologia, Imunologia e Parasitologia, Escola Paulista de Medicina, Universidade Federal de São Paulo, São Paulo 04023-062, Brazil

**Keywords:** *Escherichia coli*, gut microbiota, urinary tract infections, ExPEC, UPEC, comparative genomics, non-lactose fermenting, antimicrobial resistance

## Abstract

Extra-intestinal pathogenic *Escherichia coli* (ExPEC) may inhabit the human gut microbiota without causing disease. However, if they reach extra-intestinal sites, common cystitis to bloodstream infections may occur, putting patients at risk. To examine the human gut as a source of endogenous infections, we evaluated the *E. coli* clonal diversity of 18 inpatients’ guts and their relationship with strains isolated from urinary tract infection (UTI) in the same hospital. Random amplified polymorphic DNA evaluated the clonal diversity, and the antimicrobial susceptibility was determined by disk diffusion. One isolate of each clone detected was sequenced, and their virulome and resistome were determined. Overall, 177 isolates were screened, among which 32 clones were identified (mean of two clones per patient), with ExPEC strains found in over 75% of the inpatients’ guts. Endogenous infection was confirmed in 75% of the cases. ST10, ST59, ST69, ST131, and ST1193 clones and critical mobile drug-resistance encoding genes (*bla*_CTX-M-15_, *bla*_OXA-1_, *bla*_DHA-1_, *aac(6′)-lb-cr, mcr-1.26, qnrB4*, and *qnrB19*) were identified in the gut of inpatients. The genomic analysis highlighted the diversity of the fecal strains, colonization by lactose-negative *E. coli*, the high frequency of ExPEC in the gut of inpatients without infections, and the presence of β-lactamase producing *E. coli* in the gut of inpatients regardless of the previous antibiotics’ usage. Considering that we found more than one ExPEC clone in the gut of several inpatients, surveillance of inpatients’ fecal pathogens may prevent UTI caused by *E. coli* in the hospital and dissemination of risk clones.

## 1. Introduction

*Escherichia coli* is a microorganism that colonizes the gut microbiota of humans and other animals, mainly warm-blooded. *E. coli* strains that generally do not harm a healthy host are classified as commensals, while those that cause disease are considered pathogenic [1]. According to the source of isolation, the disease, and genotypic traits, pathogenic *E. coli* strains can be divided into two groups: intestinal pathogenic *E. coli* (IPEC) and extra-intestinal pathogenic *E. coli* (ExPEC), which cause infections in the gut and outside the intestinal tract, respectively [2,3].

ExPEC strains can also be classified in pathotypes, based on the infection type, as uropathogenic *E. coli* (UPEC), neonatal meningitis-associated *E. coli* (NMEC), human sepsis-associated *E. coli* (SEPEC), and avian pathogenic *E. coli* (APEC) [4]. Johnson & Russo [5] pointed out that there are more than 50 virulence factors (VFs) associated with the ExPEC pathogenicity; however, a set of five genes (*pap, iuc/iut, afa/dra, sfa,* and *kpsMTII*) allows the characterization of strains that carry intrinsic virulence and can cause extra-intestinal infections on healthy subjects [6]. Likewise, Spurbeck et al. [7] described four genetic markers (*vat, fyuA, chuA,* and *yfcV*) that can be used to track strains that bear uropathogenic potential and, therefore, can cause urinary tract infections (UTIs).

UTI is the most common bacterial infection among humans, affecting around 150 million people yearly, mainly women. It is estimated that 70% of women will have UTIs at some point in their life, and 30% will experience recurrent infections [8,9,10]. *E. coli* is responsible for 80 to 90% of UTIs, being the main bacterium isolated in clinical samples; therefore, ExPEC strains are responsible for most infections caused by *E. coli* [11].

However, it is well established that ExPEC strains might be part of the gut microbiota, occurring in the gut of about 20% of the healthy population [12]. Such colonization might favor extra-intestinal infection since, due to their virulence factors, ExPEC strains can colonize the perianal region, reach the urinary tract, and ascend to the kidneys, causing pyelonephritis [1,13].

Although data on the frequency of ExPEC strains in the community are available, there is a lack of information on hospitalized patients. Additionally, most data on *E. coli* colonization focusing on healthy subjects rely on analyzing one single *E. coli* colony isolated from the gut. Therefore, it is difficult to evaluate the actual diversity of *E. coli* in the microbiota of these patients. Moreover, some studies that followed up the *E. coli* colonization in healthy individuals showed that some hosts did not develop infections even after long-term colonization [14,15,16]. In contrast, others suffered from UTIs just after the colonization. Therefore, the present work aimed to evaluate the diversity and the presence of pathogenic *E. coli* strains isolated from the intestinal microbiota of inpatients with and without UTI and analyze their phylogenetic origin, presence of VFs, and antimicrobial resistance (AMR). Additionally, the genomes of the fecal strains were compared with those of the *E. coli* strains isolated from the inpatients’ UTIs and strains from diverse sources (animal, clinical and environmental samples) isolated worldwide.

## 2. Materials and Methods

### 2.1. Patients, Sample Collection, and Strain Identification

Hospitalized patients of one single health center in the countryside of Sao Paulo state, Brazil, who agreed and signed the consent form, were enrolled in the study. Any patient who agreed with the study in the admission and had a urine culture request during hospitalization (due to possible infection or routine monitoring) was included. The UTI was determined based both on clinical definitions (symptoms like fever and pain) and bacterialcouting. When the inpatient had no symptoms, and all cultures were negative, it was considered that it was not infected. The rectal swabs were collected at the same time as the urine. In cases where the patient was under antibiotic therapy, both swab and urine were collected before the therapy exchange.

Eighteen rectal swabs were collected, between June 2019 and March 2020, from 18 inpatients, one rectal swab for each patient. The rectal swab collection occurred at the first request for urine culture of the patients after their admission at the hospital. The rectal swabs were collected by the hospital staff using the COPAN 132C swabs for collection and transport in Cary-Blair gel medium (Venturi Transystem, COPAN, Brescia, Italy). The rectal swabs were streaked onto MacConkey agar (BD Difco, Sparks, MD, USA) and incubated at 37 °C for 24 h. At least 10 lactose-fermenting (lactose-positive) and all non-fermenting (lactose-negative) colonies with distinct morphologies were chosen for biochemical identification [17,18,19,20]. After biochemical evaluation, all strains were submitted to matrix-assisted laser desorption ionization–time-of-flight (MALDI-TOF) mass spectrometry using a Microflex LT (Bruker Daltonics, Billerica, MA, USA) equipment for the species confirmation as *E. coli*. Each colony and the isolates obtained from the urine were stored in Lysogeny Broth (LB), supplemented with 15% glycerol, and kept at –80 °C.

In all cases in which the UTI caused by *E. coli* was confirmed by the hospital’s clinical microbiology laboratory and was timely linked with the rectal swab collection, a single colony of *E. coli* obtained from the urine culture was sent to us for further analysis. *E. coli* strains isolated from UTI were evaluated only when the infection occurred simultaneously with the rectal swab collection. At our laboratory, we confirmed purity and species using biochemical identification followed by MALDI-TOF.

The strains were identified with an arbitrary number to link all strains obtained from each patient, followed by the letter F when isolated from rectal swabs, while the isolates from the urine were identified with the letter U.

### 2.2. Clonal Analyses of the Isolates

All *E. coli* isolates from the gut and urine were assessed by random amplified polymorphic DNA (RAPD) to determine the clonal relationship of the strains as proposed by Nielsen and collaborators [21]. This assessment was performed in two distinct PCRs, each using a single primer, 1247 (5′-AAGAGCCCGT-3′) and 1283 (5′-GCGATCCCCA-3′), as previously described [21]. The PCRs were done using GoTaq^®^ Green Master Mix (Promega, Madison, WI, USA) with 10 pmol of primer, 1,5 µL of DNA template obtained from bacterial heat-lysates, and sterilized water for PCR. The cycle conditions for the 1247 primer were: 95 °C for 5 min; 35 cycles (95 °C for 1 min, 38 °C for 1 min, 72 °C for 2 min); 72 °C for 10 min; and for the primer 1283 were: 95 °C for 5 min; 35 cycles (95 °C for 1 min, 36 °C for 1 min, 72 °C for 2 min); and 72 °C for 10 min extension.

All fecal isolates from each inpatient were evaluated in the same agarose gel, including the urine isolate when there was one. The clones were determined based on the amplification pattern of the samples obtained by PCR as previously described [22], comparing only the strains isolated from the same patient. Accordingly, when the amplification profile of the isolates in both PCRs was the same, the isolates were identified as clones; if the amplification profiles differed in up to two divergent bands in one of the PCRs and had an identical pattern in the other, the isolates were identified as a subclone. When three or more bands distinguished the isolates in any PCR, the isolates were identified as distinct clones [22]. All distinct clones and subclones obtained from each patient were kept and used in the remaining experiments.

### 2.3. Whole-Genome Sequencing and Genomic Analyses

All distinct clones and subclones detected by the clonal analysis and five *E. coli* strains isolated from urine were de novo sequenced. MicrobesNG (Birmingham Research Park, Birmingham, UK) sequencing service performed the genome extraction, library preparation, genome sequencing, and assembly. The DNA was extracted and quantified using the Quant-iT dsDNA HS assay kit and the plate reader AF2200 (Eppendorf, Germany). The genomic libraries were prepared using Nextera XT Library Prep Kit (Illumina, San Diego, USA), with 2 ng of DNA, with 1 min and 30 sec of elongation. The libraries were sequenced using the Illumina platform to get paired reads of 2 × 250 bp. The reads’ adapters were trimmed using Trimmomatic 0.30 with a quality cutoff of Q15 [23]. The mean coverage of the genomes was 68.4× (all above 34.7×), and NCBI SRA analyses pointed out that most of the reads provided for each genome had a Phred quality score of 37. Genomes were assembled using SPAdes software (version 3.7), and contigs were annotated using Prokka (version 1.11). The SRA and the assembled genomes of the 49 *E. coli* were deposited at NCBI. Genome analyses were conducted as previously published [24] in the Center for Genomic Epidemiology (CGE) using services of identification of acquired virulence genes (VirulenceFinder version 2.0) [25], serotype (SerotypeFinder version 2.0) [26], antibiotic resistance genes (ResFinder version 4.1) [27,28], plasmids (PlasmidFinder version 2.0) [29], presence of *fumC* and *fimH* typing (CHTyper version 1.0) [30], and sequence type determination (MLST version 2.0), following the Warwick scheme [31,32,33]. The AMR genes were confirmed using the National Database of Antibiotic-Resistant Organisms (NDARO) [34].

Phylogenetic group identification was performed using ClermonTyping [35]. Phylogenetic trees were built using the Codon-tree method evaluating the protein and nucleotide sequences of 1000 single-copy CDS in the RAxML matrix at the Bacterial and Viral Bioinformatics Resource Center (BV-BRC–formerly Pathosystems Resource Integration Center–PATRIC) [36]. The trees’ final layout was built using iTOL (version 6) [37].

Specific sequence type trees (ST131, ST69, ST10, and ST1193) were built using additional genomes identified at PATRIC and the Similar genome finder service to each strain as previously published [24]. A *P*-value threshold of 0.001 and a distance of 0.01 were used as search parameters against all *E. coli* public genomes available in the database in June 2021.

### 2.4. Evaluation of Antimicrobial Susceptibility

The selected clones were tested for antimicrobial susceptibility by the disc-diffusion method on Mueller Hinton agar (Oxoid, Basingstoke, UK) [38]. The test was performed for 12 antibiotics, which are commonly used for UTI treatment, including amikacin (30 µg), gentamicin (10 µg), imipenem (10 µg), ertapenem (10 µg), meropenem (10 µg), tigecycline (15 µg), cefoxitin (30 µg), ciprofloxacin (5 µg), ceftazidime (10 µg), cefotaxime (5 µg), aztreonam (30 µg), and cefepime (30 µg). Resistance to polymyxin B was screened by the drop test with polymyxin B (4.0 µg/mL) [39].

The results were interpreted using Brazilian Committee on Antimicrobial Susceptibility Testing (BrCAST/EUCAST) breakpoint guidelines (version 11.0) [40,41]. *E. coli* (ATCC 25922) and *Pseudomonas aeruginosa* (ATCC 27853) were used as quality control strains for the test.

Additionally, the agar dilution method was performed on strains identified with an Extended Spectrum β-Lactamase (ESBL) phenotype to determine their minimal inhibitory concentrations (MICs). Amikacin, gentamicin, tigecycline, ciprofloxacin, levofloxacin, imipenem, ertapenem, meropenem, aztreonam, ceftriaxone, ceftazidime, cefepime, and ampicillin/sulbactam were used. Furthermore, strains screened as resistant to polymyxin B by the drop test had the MIC determined for polymyxin B and colistin by broth microdilution. The results were evaluated according to BrCAST/EUCAST guidelines as described above [41].

The strains were defined as MDR when they presented resistance to at least one antibiotic of at least three classes [42].

### 2.5. Ethical Approval

This study was conducted following the World Medical Association’s Declaration of Helsinki. The research project was reviewed and approved by the National Research Ethics Commission (CONEP) of the Brazilian National Health Council (CNS) [CAAE number: 06258319.2.0000.5505, and CEP number: 0048/2019] with the agreement of the Research Ethics Committee of Federal University of São Paulo (UNIFESP) and Irmandade Santa Casa de Misericordia de Mogi Guaçu. The patients provided their written informed consent to participate in this study.

## 3. Results

### 3.1. Clonal Diversity of E. coli Strains from the Gut of Inpatients

Eighteen inpatients were evaluated in the study (15 women and three men), and 12 were under antibiotic therapy during the collection of the rectal swabs. Among the evaluated patients, 13 had UTIs; according to the microbiological analyses performed by the hospital’s clinical microbiology laboratory, 12 UTIs were due to *E. coli*, and one was due to *Klebsiella oxytoca*. A total of five patients did not have infections during the swab collection. The mean age of the patients was 64.7 years, ranging from 21 to 88 years (Table 1). 

Overall, 196 colonies obtained from rectal swabs were screened, and 177 were confirmed as *E. coli* (154 lactose-positive and 23 lactose-negative). It was not possible to recover any facultative Gram-negative bacteria from the rectal swab of one patient (P15).

**Figure 1:**
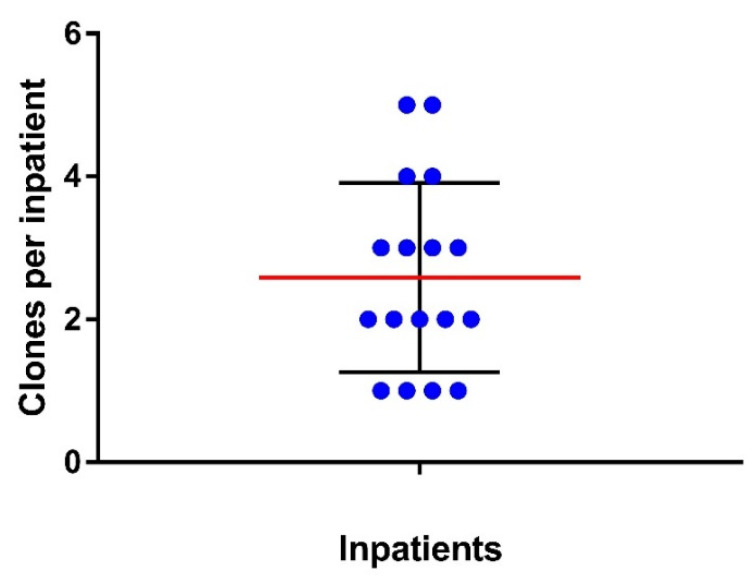
*Escherichia coli* clonal diversity identified by RAPD for 17 patients. Each dot represents one patient; the red line indicates the means of clones per patient, with standard deviation (SD), in black lines.

All colonies were assessed by RAPD to determine each inpatient’s *E. coli* clonal diversity. The results showed that most inpatients had a heterogeneous composition of *E. coli* in their intestines, with clonal diversity ranging from one to five different clones per inpatient (Figure 1). Moreover, approximately 59% of the inpatients carried two or more clones. All 44 diverse isolates (34 clones and 10 subclones) found among the 17 patients were sequenced.

The strains isolated from UTIs were also tested by RAPD and compared with the fecal strains. Among them, nine were identical to fecal strains from the same inpatient (seven clones and two subclones).

### 3.2. Phylogenetic Classification and Multilocus Sequence Types among Sequenced E. coli Clones

**Figure 2:**
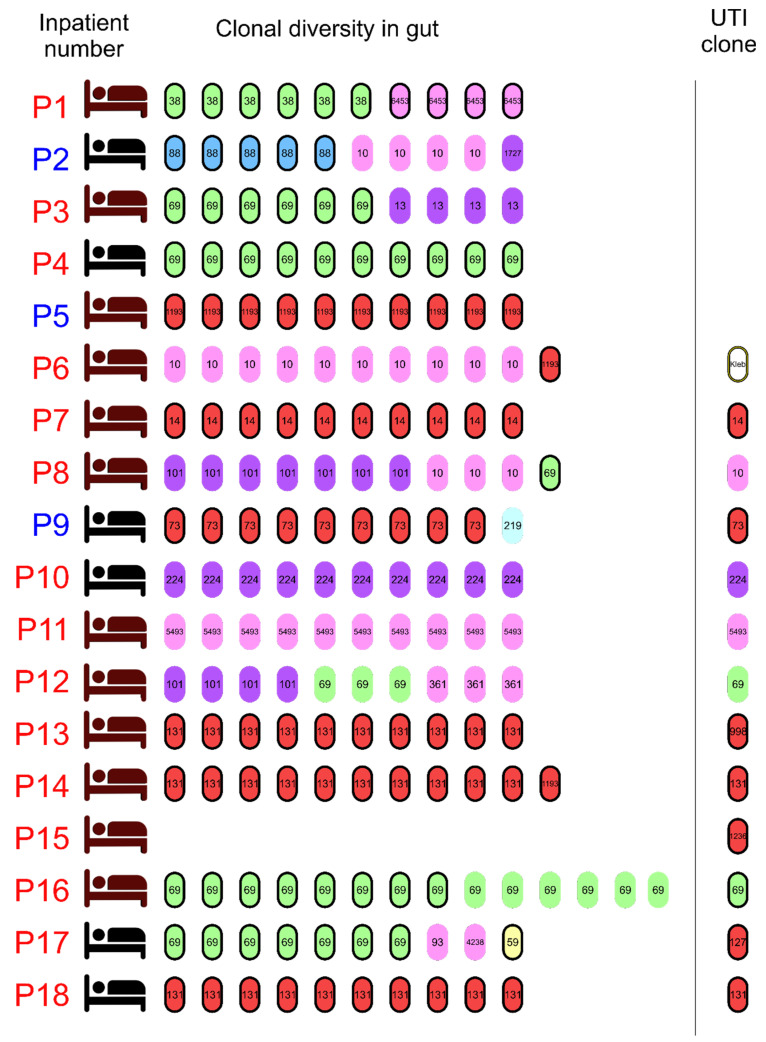
Graphic scheme of the distribution of all *Escherichia coli* clones assessed in the study. Each bacillus represents one of the colonies evaluated. The bacilli colors represent the phylogenetic origin of the strain as follows: pink, phylogroup A; purple, phylogroup B1; light red, phylogroup B2; blue, phylogroup C; green, phylogroup D; light blue, phylogroup E; and yellow, phylogroup F. The bacilli’s thick black outline indicates that the bacterium was classified as ExPEC+ or UPEC+. The number inside the bacillus represents the ST. The color of the inpatient text represents the patient’s gender, blue for male and red for female. The bed colors represent the usage of antibiotics during the swab collections, with brown beds representing inpatients under antibiotic therapy and black beds representing inpatients who were not using antibiotics. The *Klebsiella oxytoca* isolated from the urinary tract infection of one inpatient is represented by a white bacillus with a yellow edge and the word “Kleb” written inside it. No Gram-negative, aerobic, facultative bacteria were detected in the feces of patient P15.

The genomic analyses confirmed the *E. coli* diversity except for two strains assigned as distinct clones by RAPD, which were subclones of two different clones (SMG002F6 and SMG003F3); therefore, 32 clones were found in the inpatients. Evaluation of the phylogenetic origin of the strains pointed out the presence of seven phylogroups, and the phylogroups B2 and D were the most frequent in the population studied (eight strains, 25% each) (Figure S1). About 47% of the inpatients (eight inpatients) were colonized by strains from two or more phylogenetic origins, and three inpatients were colonized by different clones from the same phylogroup (Figure 2).

**Figure 3:**
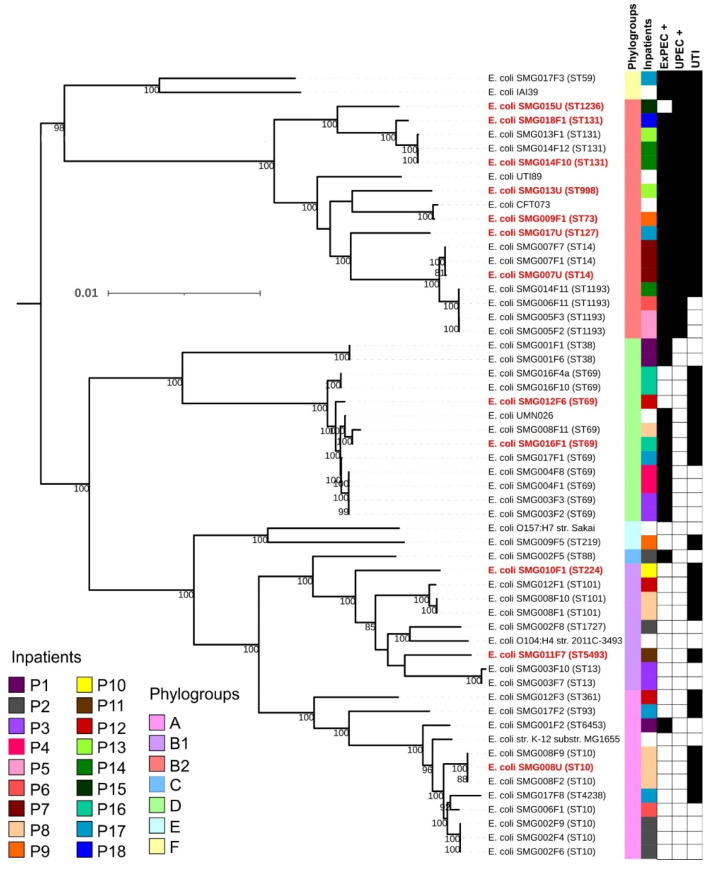
Genomic relationship of the 49 sequenced strains. In red and bold are identified clones identical to the urine strain or the urine strain itself. From the inside out, the columns represent the phylogenetic group, inpatients’ identification, strains classified as ExPEC+ (presence of at least two among the virulence markers: *pap, sfa, afa, iuc/iut,* and *kpsMTII*), strains classified as UPEC+ (presence of at least three of the *vat, yfcV, chuA,* and *fyuA* genes), and patients’ conditions regarding the presence of urinary tract infection (UTI). Filled squares indicate positivity.

The MLST of the 32 distinct clones revealed the presence of 19 STs. ST69 was the most frequent, representing 21.8% of the clones (seven strains) and being present in the intestinal tract of six inpatients (35%). The ST131, ST10, and ST1193 occurred in three inpatients each, and ST101 in two inpatients; the remaining STs occurred only once (Figure 2 and Figure 3). Strains belonging to the ST10 clonal complex (ST10cc) were found in five inpatients, including the three ST10 strains already mentioned (Figure 3).

A phylogenetic tree built with the 44 strains isolated from the gut (32 clones and 12 subclones) and five strains isolated from UTI (two subclones and three that were not identified in the inpatients’ microbiota) highlighted the clonal diversity of the evaluated population, a genetic relationship among some strains isolated from different inpatients, and the high frequency of strains belonging to the ST69 in the population evaluated (Figure 3).

Additional phylogenetic trees focusing on the most frequent STs detected in the present study (ST69, ST10cc, ST131, and ST1193) highlighted the closest relationship among the strains isolated from the microbiota identified here, either related or not to UTI, and strains isolated from extra-intestinal infections worldwide.

The ST131 tree evidenced that the three strains were not directly related; instead, they were grouped into three subclusters (Figure S2). In fact, additional analyses of the *fimH* gene confirmed that SMG014F10 belonged to the ST131-H30-Rx subclone, SMG013F1 to ST131-H30-R, and SMG018F1 to the subclone which did not have the *fimH* gene (H0) located very far in the phylogenetic tree than the other two strains.

The ST69 strains isolated from inpatients P3 and P4 were more related to each other than those isolated from patients P8 and P16. The ST69 strains were found in five different clusters, and it is important to point out that both ST69 strains isolated from patient P16 (SMG016F1 and SMG016F10) belonged to different clusters (Figure S3), showing that some hosts can be colonized by different and unrelated clones belonging to the same ST.

Three patients were colonized by strains belonging to ST1193 (P5, P6, and P14), with the strains isolated from patients P6 (SMG006F11) and P14 (SMG014F11) being closely related. NIH pathogen detection tool found a difference of 35 SNPs among these two strains and showed that they belong to the pathogen SNP cluster PDS000047048 with more than 88 strains in October 2022, which were isolated from blood and urine in diverse countries worldwide. In contrast, the strains isolated from patient P5 (SMG005F2 and SMG005F3) belonged to a different cluster (Figure S4) and were subclones with 13 SNPs. Although there is no information on whether these patients were hospitalized in the same area, the rectal swabs of inpatients P5 and P6 were collected in the same month. In contrast, the sample from patient P14 was collected two months later. The evaluation of the genome of strains isolated worldwide and displaying a similar genome to these three strains showed that most were isolated from extra-intestinal infections, mainly UTIs and bloodstream infections.

All strains of the ST10cc identified in our study belonged to different clusters (Figure S5) and were unrelated. Additionally, the isolation source of the other strains from ST10cc included in the evaluation was diverse, including animals’ guts, water, and food.

### 3.3. Virulence Profile, Intrinsic Virulence, and Uropathogenic Potential of E. coli Strains from the Gut and Urine

The virulome of the strains was assessed, and strains were molecularly classified as ExPEC+ (presence of two or more out of the *pap, sfa, afa, iuc/iut,* and *kpsMTII* genes) or UPEC+ (presence of ≥ three of the *vat, yfcV, chuA,* and *fyuA* genes) according to their genotype. Overall, 14 inpatients (82.4%) were colonized by one or more *E. coli* strains classified as ExPEC; eight of these 14 inpatients also harbored strains bearing molecular markers to be classified as potentially uropathogenic. All patients who did not have UTIs and the patient with UTI due to *K. oxytoca* were colonized by strains classified as ExPEC (Figure 2).

The 17 clones classified as ExPEC+ and isolated from the 14 inpatients belonged to nine STs (ST14, ST38, ST59, ST69, ST73, ST88, ST131, ST1193, and ST6453). The three inpatients not colonized by strains classified as ExPEC+ or UPEC+ had UTI. Patients P10 and P11 had one single clone each in the gut (ST224 and ST5493, respectively), which was the same that caused their UTIs. The remaining patient (P12) was colonized by three *E. coli* clones, including one ST69 clone, which was responsible for the UTI.

The 12 *E. coli* strains isolated from the inpatients’ urine were different clones as determined by their STs, CH type, serotypes, and proximity in the phylogenetic tree (3). These strains belonged to ST10 (11-215, O101:H10), ST14 (14-37, O15:H5), ST69 (35-27, O17:H18, and 35-27, O153:H2), ST73 (24-102, O6:H1), ST127 (14-2, O6:H31), ST131 (40-30, O25:H4, and 40-0, O25:H4), ST224 (4-61, O9a:H30), ST998 (52-76, O2:H6), ST1236 (195-253, O68:H6), and ST5493 (4-396, O173:H7).

Among the nine *E. coli* strains that colonized the gut and caused the UTI, four (~ 33%) harbored few VFs and did not fulfill the molecular criteria to be classified as ExPEC+ or UPEC+. The remaining three strains had diverse VFs, including those related to the ExPEC+ and/or UPEC+ classifications (Figure 3).

Putative exogenous infections occurred due to strains from ST127 (P17), ST998 (P13), and ST1236 (P15), classified as ExPEC+/UPEC+ or UPEC+. Noteworthy is the fact that inpatient P17 was colonized by four different *E. coli* fecal clones, including two classified as ExPEC+, even though the inpatient had UTI by another ExPEC+ strain (ST127) not detected in his gut. Among the three inpatients with putative exogenous infections, P17 was the only one that was not under antibiotic therapy at the rectal-swab collection.

### 3.4. Gut Colonization by Multidrug-Resistant (MDR) E. coli

**Figure 4:**
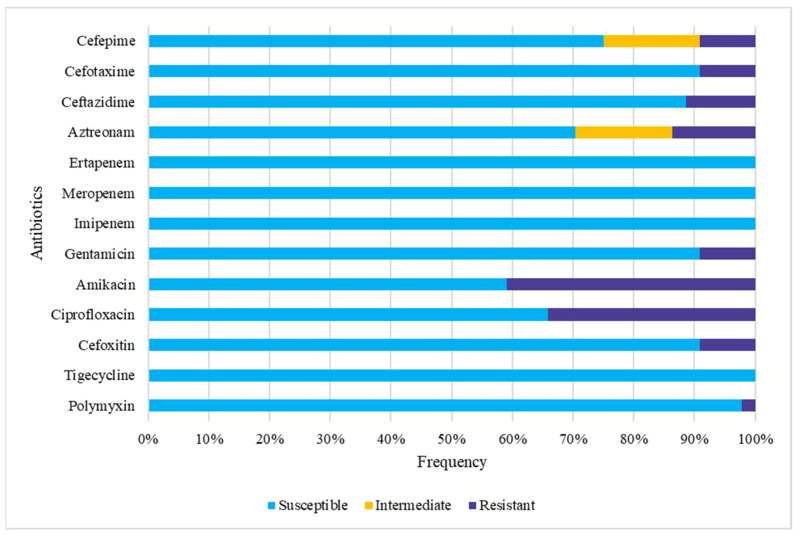
Frequency of antimicrobial resistance of fecal *E. coli* strains. Intermediate = susceptible, increased exposure.

Among the 44 fecal isolates studied, the disk-diffusion test revealed the presence of a resistant phenotype for nine of the antimicrobials tested. All strains were susceptible to carbapenems and tigecycline (Figure 4). A total of 18 strains (40.9%) were resistant to amikacin and 15 (34.1%) to ciprofloxacin (Figure 4). A total of 17 strains (38.6%) were susceptible to all antimicrobials tested (Table S1). Altogether, 13 patients (76.5%) were colonized by at least one *E. coli* that displayed a resistant phenotype to at least one antimicrobial, and MDR strains were found colonizing the gut of nine patients (52.9%). In five patients (29.4%), all *E. coli* strains were susceptible to all the antimicrobials assessed.

Eleven fecal strains were classified as MDR, with four of them (two clones and two subclones) (SMG008F2, SMG008F9, SMG014F10, and SMG014F12) being classified as ESBL producers. One strain (SMG012F6) was resistant to polymyxin, and another was a putative AmpC producer (SMG010F1).

Only four of the 12 strains (33.3%) isolated from UTI showed resistance to at least one of the antibiotics tested by the disk-diffusion test. Three of them displayed the MDR phenotype, including ESBL producers.

The fecal ESBL (SMG008F2, SMG008F9, SMG014F10, and SMG014F12) and AmpC (SMG010F1) producers and their corresponding clones isolated from urine (SMG008U, SMG010U, and SMG014U) were evaluated by agar dilution (Table 2). The results showed that these strains have resistant phenotypes displaying high MIC values, although the subclones presented different resistance levels to some antibiotics (Table 2). Considering the disk-diffusion assay result, the strain SMG010F1 was resistant only to ceftazidime, cefoxitin, and amoxicillin with clavulanic acid. Still, it displayed MIC values equal to or higher than 32 µg/mL for all cephalosporins evaluated in the agar dilution assay (Table 2).

Considering the genomic content, 22 of the 49 strains sequenced harbored at least one AMR gene (Table S2). A total of 35 AMR genes were identified among the strains, with *bla*_TEM-1B_ being the most frequent, occurring in 10 strains. Punctual mutation in the *gyrA* gene, which confers resistance to quinolone (*gyrA* p.S83L and/or *gyrA* p.D87N), occurred in 11 strains.

The ST131 strains from the H30 cluster (SMG013F1 and SMG014F10) presented two punctual mutations in the *gyrA* gene (p.S83L and p.D87N), and SMG014F10 also harbored additional genes that confer resistance to aminoglycoside (*aac(3)-IIa*, *aac(6′)-Ib-cr, aph(3′)-Ib, aph(6″)-Id*), β-lactam (*bla*_CTX-M-15,_ *bla*_TEM-1B_, *bla*_OXA-1_), sulfonamide (*sul2*), tetracycline (*tet(B)*), and phenicol (*catA1*, *catB3*) (Table S2).

Regarding the mobile AMR genes of concern, *E. coli* strains bearing *aac(6′)-Ib-cr*, *qnrB1, qnrB4, qnrB19*, *bla*_CTX-M15_, *bla*_OXA-1_, *bla*_DHA-1_, and *mcr-1.26* were identified both in the gut and the urinary tract of four inpatients (Table 2 and Table S2). The strain bearing *mcr-1.26* (SMG012F6) displayed MIC values of 8 µg/mL and 4 µg/mL to colistin and polymyxin, respectively, carried the *qnrB19* gene, despite being susceptible to ciprofloxacin and levofloxacin (MIC ≤ 0.06 µg/mL for both fluoroquinolones).

## 4. Discussion

*E. coli* is an important etiological agent of human extra-intestinal infections, and the human gut is a natural reservoir and source of ExPEC [5,10,43,44]. Therefore, understanding the diversity and the frequency of ExPEC strains that colonize the gut of hospitalized patients may help implement measures to prevent *E. coli* infections and the dissemination of pathogenic and resistant strains through the hospital environment.

To enlarge the knowledge regarding the gut reservoir, the present work focused on accessing the clonal diversity of *E. coli* strains that colonized the gut of inpatients with and without UTI caused by *E. coli* and the presence of VFs and AMR genes. For this purpose, at least ten colonies of each inpatient, including lactose-negative isolates, obtained from rectal swabs plated on MacConkey agar were evaluated.

After evaluating up to 20 *E. coli* colonies from rectal swabs or stools, Mosavie et al. (2019) concluded that ten colonies were sufficient to access the *E. coli* clonal diversity in the gut of hospitalized patients [45]. However, the authors used chromogenic agar to guide their evaluation, which generally excludes strains that lack the typical *E. coli* biochemical behavior (e.g., lactose fermentation) [24]. Therefore, to better evaluate the *E. coli* diversity in the inpatients’ guts, we screened ten lactose-positive and all the lactose-negative colonies available per patient, reaching up to 14 colonies when the patient harbored a mix of lactose-positive and lactose-negative strains. We recovered at least one *E. coli* clone in 94.6% of the patients (17 out of 18). The exception was a single patient colonized by gram-positive bacteria. We did not recover anaerobic-facultative gram-negative bacteria from the rectal swab of this patient despite the usage of diverse media. For the others, the evaluation of lactose-negative colonies enabled the identification of additional *E. coli* clones.

In general, studies that assess *E. coli* strains from the gut often evaluate only one representative colony from the gut environment or exclude all lactose-negative strains or both [46,47,48,49,50]. These approaches impact the evaluation of the *E. coli* strains’ diversity and exclude some important pathogens. Commonly avoided in epidemiological studies that track *E. coli* in the environment or intestinal tract [6,50,51], the lactose-negative colonies represented almost 13% of the strains evaluated in our study, being present in the gut of five inpatients (29.4%), including two, who were colonized exclusively by lactose-negative strains (P5 and P13).

Despite being continually ignored in studies that access gut colonization or other reservoirs, lactose-negative *E. coli* are important pathogens isolated from extra-intestinal sites, responsible for outbreaks, community-acquired and healthcare-associated infections in humans [24,52,53,54,55], and diverse types of infections in animals [56]. Some successful lactose-negative clones are disseminated and represent emerging high-risk clones as strains from ST1193, ST648, and many members of the ST59 and ST14 clonal complexes [24,55,57]. Interestingly, all strains from the inpatient P13 belonged to ST131 H30-R and were lactose-negative, although strains from ST131 are usually lactose-positive, as found in patients P14 and P18. These points reinforce the necessity of not neglecting *E. coli* which present an atypical biochemical phenotype when accessing a putative reservoir in epidemiological and one-health studies.

Most inpatients evaluated presented more than one type of *E. coli* strain in the gut, and the clonal diversity ranged from 1 to 4 per patient, regardless of the occurrence of UTI. In most inpatients, the *E. coli* clones identified belonged to diverse phylogenetic groups, and a combination of up to three distinct phylogroups was found. Phylogroups A, B2, and D occurred in seven of the 17 patients evaluated (41.2%). 

In recent years, the number of reports showing the diversity of *E. coli* clones in the gut increased [21,44,58,59,60]. However, most of these reports focused on describing the MLST of MDR strains isolated using a selective medium or focused on the phylogenetic origin or the presence of specific VFs. Still, these features were rarely assessed altogether.

Stoppe et al. [51] evaluated 39 studies carried out in 24 countries regarding the phylogenetic origin of *E. coli* strains colonizing the humans’ guts and showed that phylogroup A was the most frequently identified in 22 studies conducted in diverse cities around the world, including two studies in Sao Paulo, Brazil [51,61]. On the other hand, phylogroup D was the most frequent only in three studies [62,63,64] and shared the first position with phylogroup A in another [65]. In the studies conducted in Sao Paulo, phylogroups A and D showed similar frequencies and were the most frequent in two different counties [51,61]. In our study, phylogroups A, B2, and D were found in similar frequencies, colonizing the gut of 41.2% of the subjects.

Differences in the frequency of the phylogroups found may be explained by distinct approaches used to select and evaluate strains from the gut (*e.g.,* single versus multiple colonies or triplex [66] versus quadruplex [67] PCR methods) or by a phylogroup replacement of the *E. coli* population in the microbiota of the studied community. Studies carried out in Paris, with strains isolated over 30 years, showed that phylogroup B2 surpassed phylogroup A in prevalence over time in that community [68]. Nevertheless, using quantitative PCR to evaluate the phylogeny directly from feces, Smati et al. [69] showed that the proportion of the phylogroups varies in human subjects, and some phylogroups commonly underrepresented colonize the human gut in the same frequency as those called “dominant clones” evaluated when the single-colony method was applied. Additionally, in agreement with our findings, they have shown that 69% of the subjects carried two or three *E. coli* strains from different phylogroups [69].

The importance of phylogroup D in the present study was due to the high frequency of strains from ST69; six out of seven patients colonized by phylogroup D harbored at least one ST69 strain. Interestingly, all but two strains from ST69 had a diverse clonal origin, virulence, and serotype, suggesting the dissemination of different ST69 clones colonizing people in the community rather than the dissemination of one clone in the hospital environment. One of the clones, present in the gut and urine of one patient (P12), carried *mcr-1.26* and *qnrB19* genes and displayed a resistant phenotype to colistin and polymyxin but was susceptible to ciprofloxacin and levofloxacin. Strains from ST69 have been reported as a frequent pathogen in human extra-intestinal infections related to community-acquired UTI sporadic cases and outbreaks worldwide [70,71,72,73,74]. In Brazil, it has been often reported in epidemiological studies since 2009 [75,76,77,78,79]. One survey related to colistin resistance recently identified an *E. coli* ST69 strain among clinical isolates in Sao Paulo [80]. In the referred study, the authors pointed out that the *mcr-1* gene was in an IncX4 conjugative plasmid [80], which is similar to our study since the *mcr-1.26* gene and the IncX4 plasmid replicon were found in the same contig (data not shown). However, the similarities among the strains end on this since both presented diverse virulence and AMR gene contents.

Thirty-two *E. coli* clones, isolated from the gut of 17 inpatients, were classified into 19 different STs, of which five (ST69, ST10, ST131, ST1193, and ST101) occurred more than once in different subjects. A total of 15 patients were colonized by at least one of ten STs (ST10-A, ST14-B2, ST38-B2, ST59-F, ST69-D, ST73-B2, ST88-C, ST101-B1, ST131-B2, and ST1193-B2) that are regularly reported as a cause of extra-intestinal infections and were included among the 20 STs more frequently detected around the world [70]. Corroborating with these data, in the present study, some strains from these STs caused UTI in seven patients (ST69 and ST131 in two inpatients each; and ST10, ST14, and ST73 in one inpatient each). Curiously, one inpatient was colonized exclusively by an ST131-H30R clone and had UTI by an *E. coli* from ST998, which is a rarely reported ST associated with extra-intestinal infections [72,81,82,83,84]. 

Regarding the pathogenic potential of the strains, 17 (53.1%) of them had molecular markers related to intrinsic virulence (ExPEC+), and nine (28%) had the traits that determine the uropathogenic potential (UPEC+). Therefore, these strains are potential extra-intestinal pathogens colonizing about 82% of the inpatients, including all those that did not have UTIs or UTIs caused by *E. coli*. Three of four inpatients, who were not colonized by strains harboring ExPEC or UPEC molecular markers, had UTIs of endogenous origin since strains genetically identical to the urine clone were found in their gut. Interestingly, two of the seven ST69 clones identified in the present study were devoid of the molecular markers related to the ExPEC+ or UPEC+ classifications, one of which (from serotype O17:H18) caused UTI in the patient from whom it was isolated, quite similar to the strains described as a common cause of UTI in the 1990s, when isolates from ST69 were called Clonal group A (CgA) [71,85].

The endogenous infection of inpatient P10 was due to an *E. coli* strain from ST224. This ST has been reported in extra-intestinal infections in diverse hosts [86,87,88,89]. In contrast, *E. coli* ST5493 (ST20 complex) was the single clone in the gut of inpatient P11 and was responsible for the patient’s UTI; this ST was never reported to be isolated from extra-intestinal infections. It is worth pointing out that, despite the high number of VFs already recognized as important to extra-intestinal infections [5], many strains isolated from such infections and belonging to diverse STs lack the VFs related to the molecular classification of ExPEC+ or UPEC+, but are often reported from extra-intestinal infections, such as strains from ST10, ST410, ST101, and others [70,74,90,91].

Regardless of strains not harboring important UTI-associated VFs, in the present study, ExPEC+ strains colonized the gut of all the inpatients without infection. Previous studies evaluating water, food, and gut colonization demonstrated that the occurrence of UTI after gut colonization by an ExPEC strain is a matter of time. Additionally, those ExPEC+ strains linked to water and retail food that colonize the human gut were the same ones responsible for UTIs [14,44,50,92,93].

In most inpatients studied (nine out of twelve), the origin of the UTI was endogenous, even when the *E. coli* strains were resistant to antibiotics (e.g., ESBL or AmpC producers). Therefore, the knowledge regarding the presence of pathogenic or MDR strains in the gut of patients admitted to a hospital and during their stay might help in the initial treatment or guide the prevention of the infection.

Corroborating with this suggestion, one recent study evaluating nosocomial transmission of *E. coli* and multidrug-resistant genes found that *E. coli* clones, including strains displaying an MDR phenotype, disseminate through the hospital’s wards [94]. The authors showed that some of these clones colonized the inpatients’ guts before causing bloodstream infections in some of them. Similar to our study, as the authors sequenced multiple fecal *E. coli* colonies, they concluded that most patients developed an infection of an endogenous origin [94]. Interestingly, we also found two patients (P6 and P14) that shared one *E. coli* clone belonging to ST1193, a pandemic high-risk *E. coli* clone.

Surveillance rectal swab is performed in various hospitals worldwide to monitor the gut colonization by MDR bacteria during long-term hospitalization. Diverse studies in the last year showed how these surveillances provide valuable information to clinicians [95,96], helping them define the empirical treatment for severe diseases such as sepsis and preventing post-operatory or trauma-related infections, besides informing about MDR dissemination through colonization [97,98,99]. The current problem is how to refine the methods to enable the search of multiple pathogens, including non-MDR, to prevent infection in intensive care units and hospital wards.

Unfortunately, the lack of additional information and follow-up of the inpatients during their stay limited the understanding of the gut microbiota change during the hospitalization or our knowledge concerning any extra-intestinal infection they may have acquired after the rectal swab collection.

Overall, there was no significant difference in the number of *E. coli* clones in the gut when comparing patients with and without UTI (*p* > 0.05). Considering the previous use of antibiotics by 66% of patients before the rectal swab collection, we evaluated the impact of this usage on the clonal diversity and occurrence of drug-resistant *E. coli* strains. We did not identify differences in the number of clones or the presence of drug-resistant strains between the treated and untreated inpatients (*p* > 0.05). Importantly, our sample size is small, and our study focused only on evaluating *E. coli* clones; therefore, any conclusion regarding this observation is speculative. Additional studies conducted with larger samples are required. Moreover, other factors, such as the type of antibiotic used and the presence of other drug-resistant species, must be evaluated for specific conclusions in this regard.

Antimicrobial resistance is an increasing problem worldwide, and resistance to 3^rd^ generation cephalosporin (3GC) in Enterobacteriaceae is critical. In this study, the three 3GC-resistant strains found in the gut were also responsible for the UTI of the inpatient from whom they were isolated. In total, six strains (four fecal and two from urine) were β-lactamase producers (*bla*_CTX-M-15_), and two strains (one fecal and one from urine) were AmpC producers (*bla*_DHA-1_). These strains displayed high resistance levels to all cephalosporins assessed in the agar dilution assay. The strains isolated from different inpatients were not clonally linked. The β-lactamase producers belonged to ST10 and ST131 (phylogroups A and B2, respectively), while the AmpC producers belonged to ST224 (phylogroup B1). A recent One Health study in Brazil showed that β-lactamase producers from ST10 and ST131 were high-risk clones frequently isolated from humans [89]. Interestingly, in the samples evaluated, they did not find a significant presence of ST69 strains, which was the most frequent ST in the present study, nor the presence of *bla*_DHA-1_; however, they reported different strains belonging to ST224 bearing *bla*_CTX-M-2,_ *bla*_CTX-M-8,_ and *bla*_KPC-2_, all isolated in Brazilian’s Southeast from extra-intestinal infections in humans (urine) and animals (lung) [89].

## 5. Conclusions

The approach used in the present study enabled the identification of multiple *E. coli* clones in the gut and a better understanding of the prevalence of *E. coli* isolates bearing VFs and AMR genes.

Identifying risk clones harboring numerous VFs in the gut of all patients with no UTI poses a risk to these patients and the hospital environment. MDR strains producing ESBL were found in the gut of inpatients regardless of the previous usage of antibiotics, and they caused UTIs in all patients that carried them in the gut.

Finally, considering that more than 75% of the UTIs were endogenous and the high frequency of virulent and resistant strains in the gut, a surveillance program focusing on gut colonization could be used to prevent UTIs caused by *E. coli* and the dissemination of risk clones in the hospital environment.

## Figures and Tables

**Table 1:** Characteristics of the 18 inpatients studied.

Characteristics		N° (%)
Gender		
	Female	15 (83.3)
	Male	3 (16.7)
Antibiotic use		12 (66.7)
UTI agent		
	*E. coli*	12 (66.7)
	Another pathogen ^a^	1 (5.6)

^a^ Positive urine culture for *Klebsiella oxytoca*.

**Table 2:** Minimal Inhibitory Concentration (µg/mL) determination of the ESBL positive strains.

Strains	SAM	ATM	CRO	CAZ	FEP	ETP	IPM	MEM	TGC	CIP	LVX	AMK	GEN	CST	PMB
SMG008F2	**>128/4**	**64**	**256**	**128**	**64**	<0.06	<0.06	<0.06	**16**	**64**	**32**	**128**	**16**	<0.25	<0.25
SMG008F9	**>128/4**	**64**	**256**	**64**	**64**	<0.06	0.25	<0.06	**2**	**>64**	**64**	**128**	**64**	<0.25	<0.25
SMG008U	**>128/4**	**32**	**256**	**128**	**32**	<0.06	<0.06	<0.06	**1**	**>64**	**32**	8	<0.125	<0.25	<0.25
SMG010F1	**16/4**	**32**	**128**	**32**	**64**	0.25	0.125	0.125	**1**	**64**	**64**	**32**	**16**	<0.25	<0.25
SMG010U	**>128/4**	**128**	**128**	**32**	**16**	<0.06	<0.06	<0.06	0.25	**>64**	**>64**	**32**	**64**	<0.25	<0.25
SMG014F10	**>128/4**	**128**	**256**	**64**	**64**	0.125	0.25	0.125	0.25	**>64**	**32**	**16**	**32**	<0.25	<0.25
SMG014F12	**>128/4**	**128**	**>256**	**128**	**128**	<0.06	0.25	<0.06	0.5	**>64**	**64**	**16**	**128**	<0.25	<0.25
SMG014U	**>128/4**	**32**	**256**	**64**	**16**	<0.06	<0.06	<0.06	**4**	**>64**	**16**	**128**	**32**	<0.25	<0.25

SAM: ampicillin/sulbactam; ATM: aztreonam; CRO: ceftriaxone; CAZ: ceftazidime; FEP: cefepime; ETP: ertapenem; IPM: imipenem; MEM: meropenem; TGC: tigecycline; CIP: ciprofloxacin; LVX: levofloxacin; AMK: amikacin; GEN: gentamicin; CST: colistin; PMB: polymyxin B. Blue values mean susceptibility, while bold and red values mean resistance, according to BrCAST/EUCAST.

## Data Availability

The Whole Genome Shotgun project of the 49 *E. coli* strains sequenced in this study has been deposited at DDBJ/ENA/GenBank under the BioProject PRJNA701970 and accessions JAKVSS000000000-JAKVTE000000000, JAKVSL000000000-JAKVSR000000000, JAKYWU000000000, and JALEBI000000000-JALECI000000000. The versions described in this paper are JAKVSS010000000-JAKVTE010000000, JAKVSL010000000-JAKVSR010000000, JAKYWU010000000, and JALEBI010000000-JALECI010000000.

## Supplementary Materials

The following supporting information can be downloaded at: https://www.mdpi.com/article/10.3390/pathogens11121528/s1, Figure S1–Phylogenetic relationship of *Escherichia coli* strains isolated from the gut; Figure S2. Phylogenetic tree focusing on strains belonging to ST131; Figure S3. Phylogenetic tree focusing on strains belonging to ST69; Figure S4. Phylogenetic tree focusing on strains belonging to ST1193; Figure S5. Phylogenetic tree focusing on strains belonging to ST10cc; Table S1–Antimicrobial susceptibility of strains by disc-diffusion (mm); Table S2–Antimicrobial resistance genes, punctual mutations, and ST of the 49 *Escherichia coli* strains sequenced. Dataset 1–accession numbers and features of 49 *E. coli* sequenced.
