# Supplementary material for: Virulence Profile, Antibiotic Resistance, and Phylogenetic Relationships among Escherichia coli Strains Isolated from the Feces and Urine of Hospitalized Patients
Source: Pathogens. 2022 Dec 13;11(12):1528. doi: 10.3390/pathogens11121528 (PMC9782660; doi:10.3390/pathogens11121528)
Supplement: Supplementary file 1 [file pathogens-11-01528-s001.zip › Santos-Netos et al 2022 Suplementar.pdf]

Supplementary Material

Virulence Profile, Antibiotic Resistance, and Phylogenetic Relationships Among *Escherichia coli* Strains Isolated from the Feces and Urine of Hospitalized Patients

José F. Santos-Neto [1], Ana C. M. Santos [1,*], Júllia A. S. Nascimento [1], Liana O. Trovão [1], Fernanda F. Santos [2], Tiago B. Valiatti [2], Ana C. Gales [2], Ana L. V. R. Marques [3], Isabel C. Pinaffi [4], Mônica A. M. Vieira [1], Rosa M. Silva [5], Ivan N. Falsetti [3,4], Tânia A. T. Gomes [1,*]

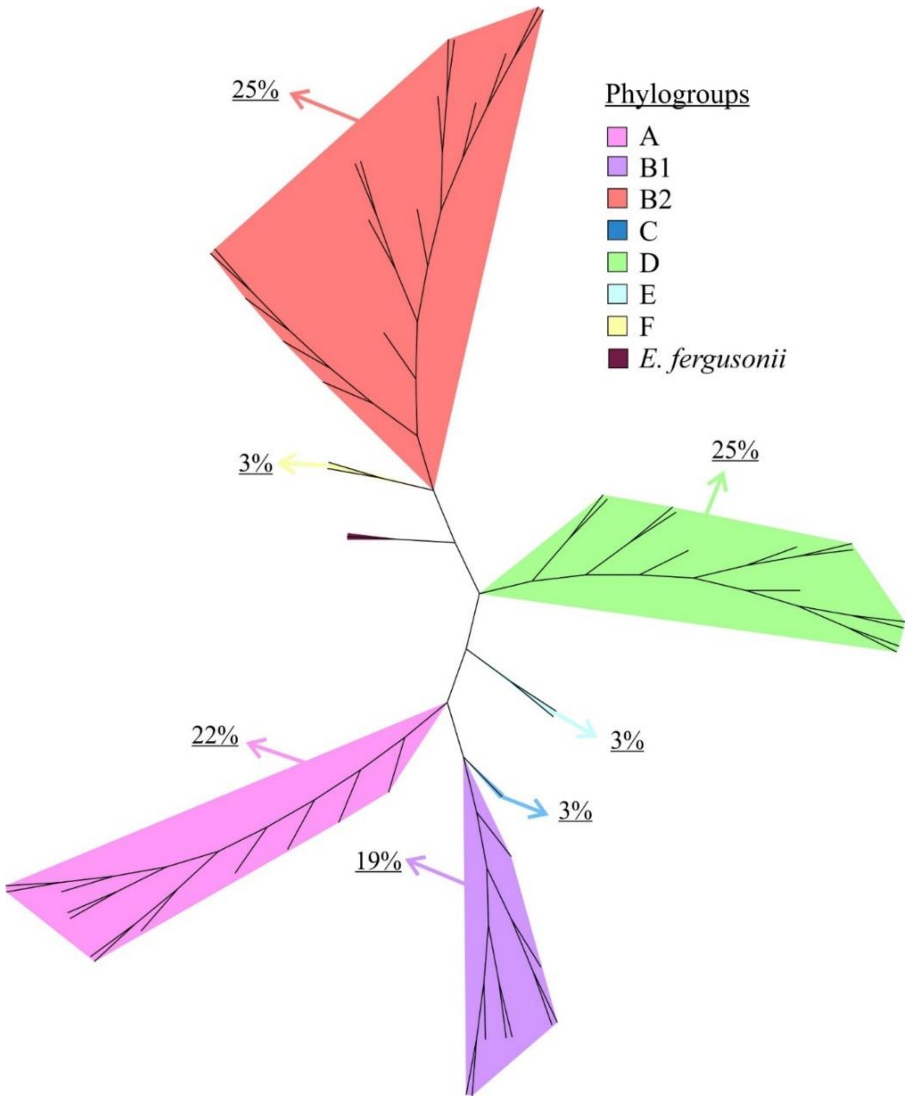

Figure S1—Phylogenetic relationship of *Escherichia coli* strains isolated from the gut. Unroot phylogenetic tree highlighting the phylogenetic origin of the 44 sequenced strains isolated from inpatients' guts. The percentages represent the frequency of each phylogroup in the sample.

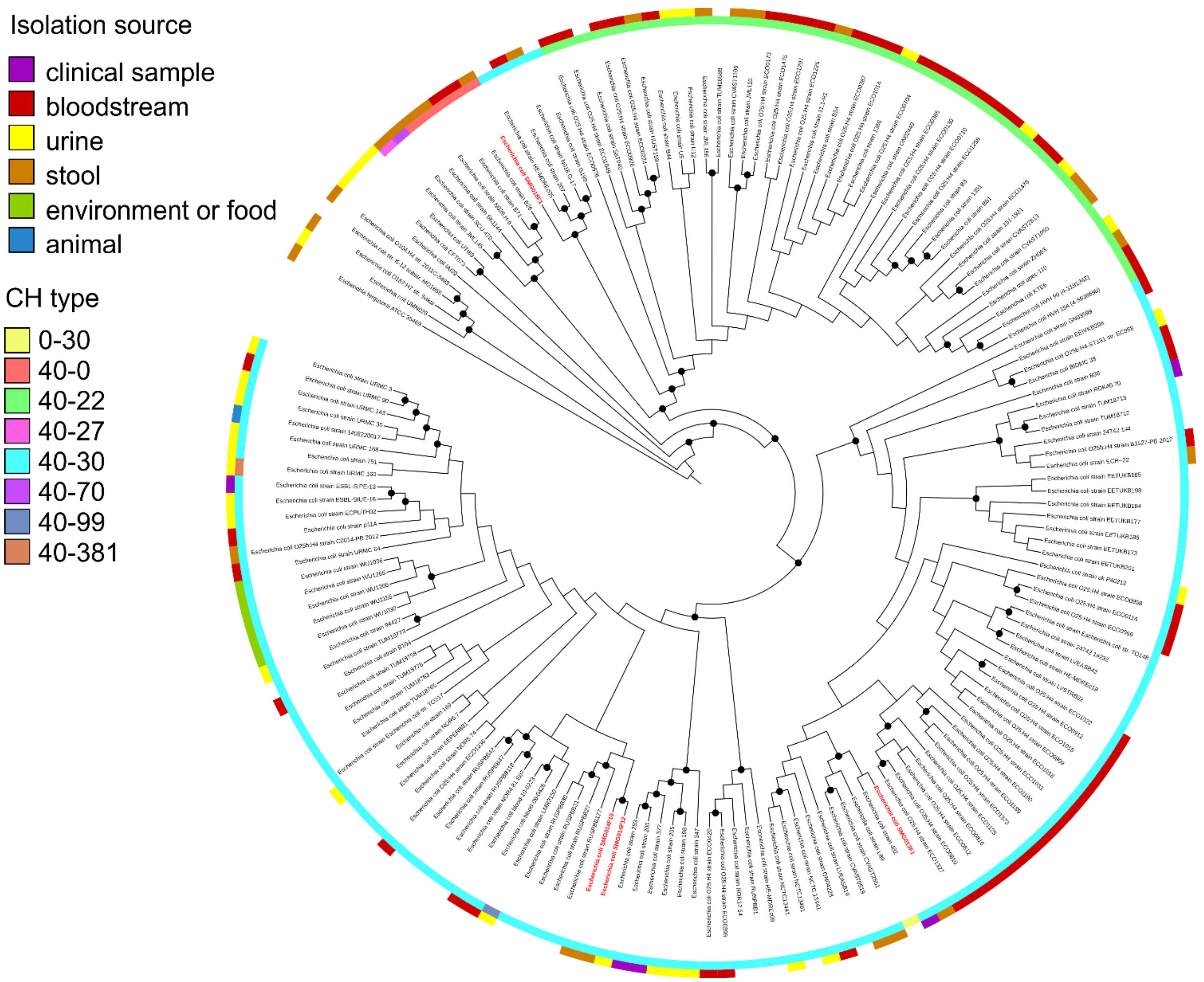

Figure S2. Phylogenetic tree focusing on strains belonging to ST131. The tree was built with 154 public *E. coli* genomes belonging to ST131, plus eight control strains (*Escherichia fergusonii* ATCC 35469, *E. coli* CFT073, *E. coli* O157:H7 str. Sakai, *E. coli* UTI89, *E. coli* str. K-12 substr. MG1655, *E. coli* UMN026, *E. coli* O104:H4 str. 2011C-3493, and *E. coli* IAI39). The strains in bold and red are those identified in the present study. The bootstrap values above 80 are indicated with a black circle on the leaf. The strain *E. fergusonii* ATCC 35469 was the root of the tree. The inner ring represents the CH type, and the outer ring is the isolation source.

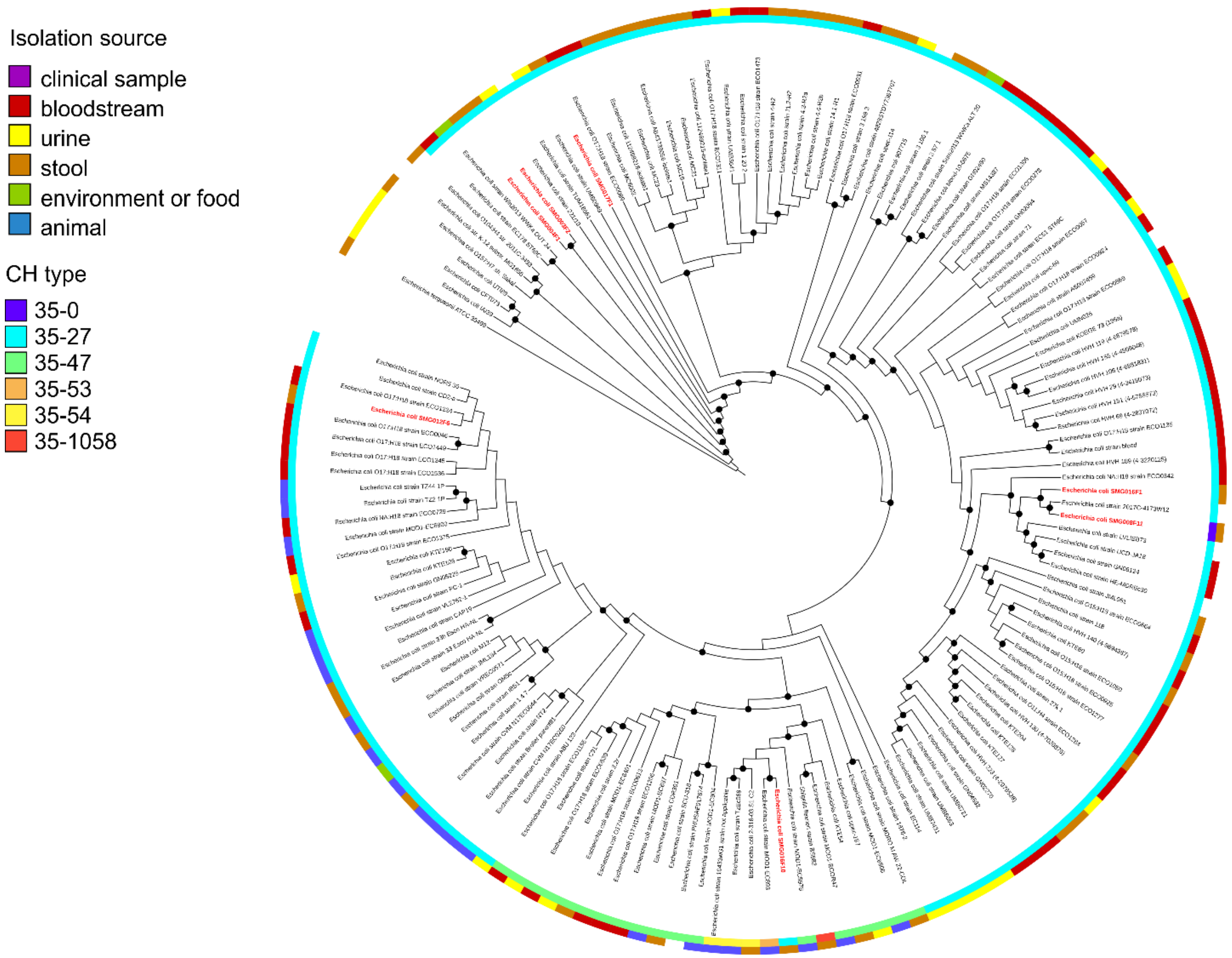

Figure S3. Phylogenetic tree focusing on strains belonging to ST69. The tree was built with 136 public *E. coli* genomes belonging to ST69, plus eight control strains (*Escherichia fergusonii* ATCC 35469, *E. coli* CFT073, *E. coli* O157:H7 str. Sakai, *E. coli* UTI89, *E. coli* str. K-12 substr. MG1655, *E. coli* UMN026, *E. coli* O104:H4 str. 2011C-3493, and *E. coli* IAI39). The strains in bold and red are those identified in the present study. The bootstrap values above 80 are indicated with a black circle on the leaf. The strain *E. fergusonii* ATCC 35469 was the root of the tree. The inner ring represents the CH type, and the outer ring is the isolation source.

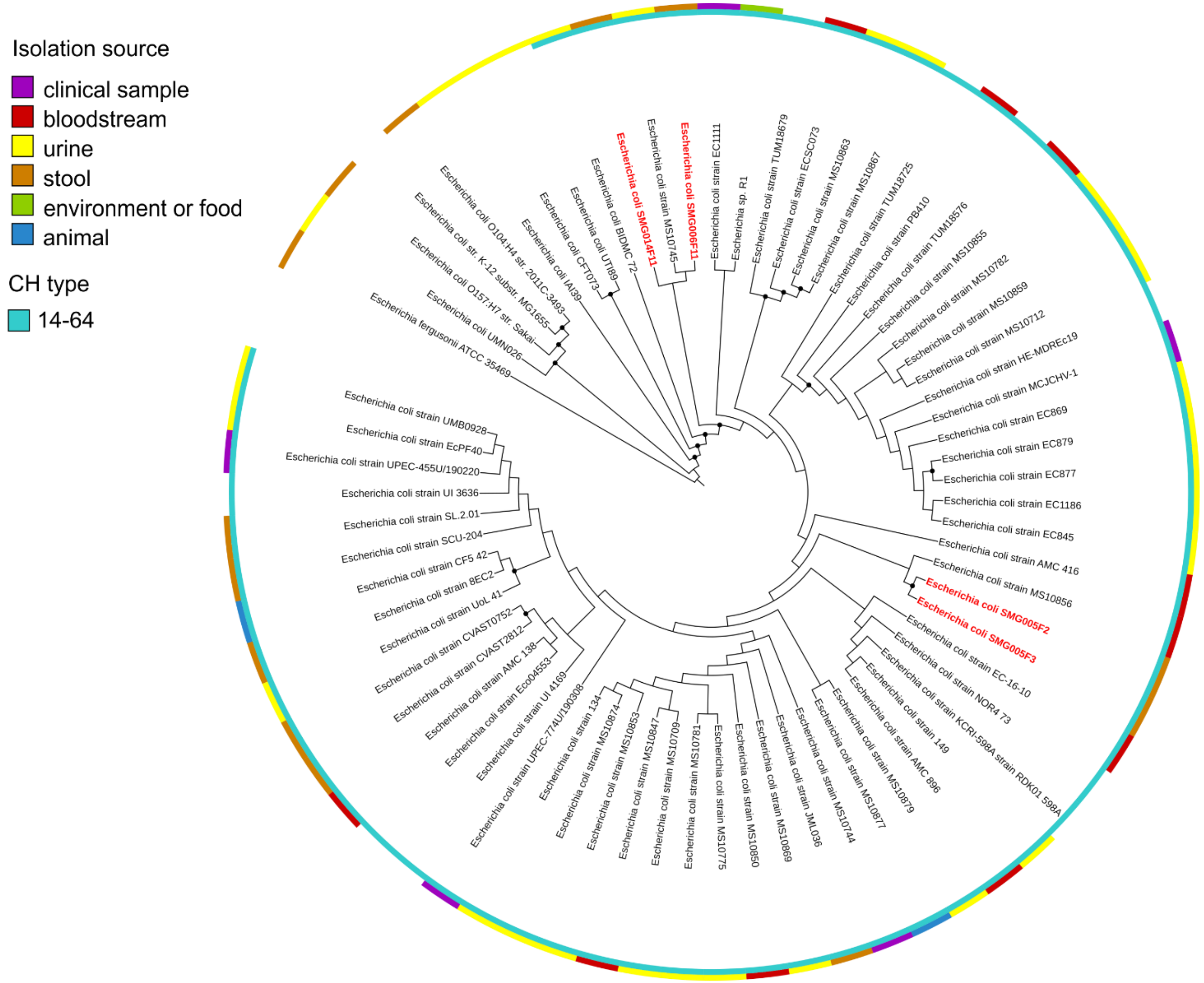

Figure S4. Phylogenetic tree focusing on strains belonging to ST1193. The tree was built with 58 public *E. coli* genomes belonging to ST1193, plus eight control strains (*Escherichia fergusonii* ATCC 35469, *E. coli* CFT073, *E. coli* O157:H7 str. Sakai, *E. coli* UTI89, *E. coli* str. K-12 substr. MG1655, *E. coli* UMN026, *E. coli* O104:H4 str. 2011C-3493, and *E. coli* IAI39). The strains in bold and red are those identified in the present study. The bootstrap values above 80 are indicated with a black circle on the leaf. The strain *E. fergusonii* ATCC 35469 was the root of the tree. The inner ring represents the CH type, and the outer ring is the isolation source.

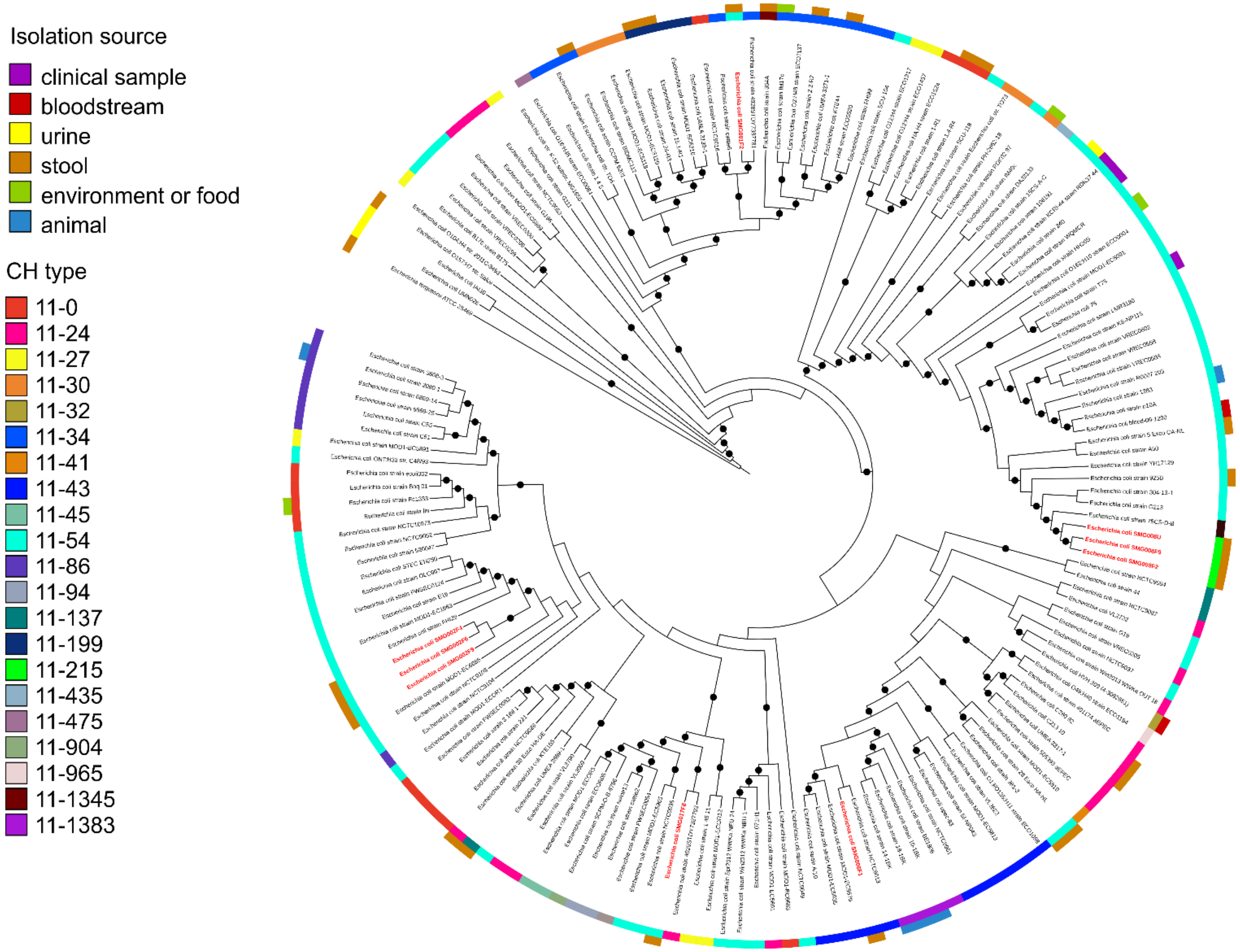

Figure S5. Phylogenetic tree focusing on strains belonging to ST10cc. The tree was built with 154 public *E. coli* genomes belonging to ST10, plus six control strains (*Escherichia fergusonii* ATCC 35469, *E. coli* O157:H7 str. Sakai, *E. coli* str. K-12 substr. MG1655, *E. coli* UMN026, *E. coli* O104:H4 str. 2011C-3493, and *E. coli* IAI39). The strains in bold and red are those identified in the present study. The bootstrap values greater than 80 are indicated with a black circle on the leaf. The strain *E. fergusonii* ATCC 35469 was the root of the tree. The inner ring represents the CH type, and the outer ring is the isolation source.

Table S1—Antimicrobial susceptibility of strains by disc-diffusion.

Strains	Antibiotics (mm)											
	AMK	GEN	IPM	MEM	ETP	TGC	CIP	FOX	ATM	CAZ	CTX	FEP
SMG001F1	**16**	17	27	30	30	22	**21**	19	25	24	23	26
SMG001F2	19	20	28	32	32	22	33	25	28	27	26	30
SMG001F6	**17**	18	28	29	30	20	27	25	26	25	25	28
SMG002F4	**17**	19	30	30	33	20	30	23	27	27	26	28
SMG002F5	20	22	32	34	30	23	**16**	25	**12**	34	>	36
SMG002F6	**16**	19	29	32	32	21	28	26	33	31	30	35
SMG002F8	20	21	30	32	34	23	32	26	29	27	29	33
SMG002F9	20	20	30	34	34	23	32	27	30	30	28	34
SMG003F2	**17**	19	29	29	31	21	**20**	22	24	22	22	28
SMG003F3	**16**	18	28	30	31	21	**21**	20	24	22	22	28
SMG003F7	20	20	30	30	32	22	23	24	27	24	26	27
SMG003F10	26	26	>	>	40	26	38	28	>	32	>	>
SMG004F1	20	23	31	33	34	23	23	24	28	24	26	31
SMG004F8	20	21	30	31	33	23	24	24	28	26	27	30
SMG005F2	18	19	28	30	33	23	**9**	26	26	26	26	25
SMG005F3	18	20	27	30	32	23	**9**	27	26	25	24	29
SMG006F1	20	20	32	32	34	23	33	25	34	31	30	34
SMG006F11	**15**	**10**	30	32	32	23	**10**	26	**9**	29	32	32
SMG007F1	21	23	30	36	36	22	32	25	30	30	>	30
SMG007F7	20	20	30	32	31	23	29	26	30	30	29	32
SMG007U	20	20	30	31	31	21	31	25	>	28	>	30
SMG008F1	18	20	29	31	31	19	26	21	24	25	25	28
SMG008F2	**14**	21	33	34	30	18	**6**	**15**	**14**	**18**	**8**	**15**
SMG008F9	**13**	19	32	33	30	18	**6**	**14**	**16**	**10**	**18**	**18**
SMG008F10	18	20	30	32	31	18	28	21	28	25	26	27
SMG008F11	19	19	26	29	30	22	30	23	27	26	24	28
SMG008U	**17**	19	26	29	28	**16**	**6**	22	**17**	**15**	**12**	**17**
SMG009F1	20	20	30	32	34	23	32	25	32	28	30	34
SMG009F5	19	20	29	32	33	22	29	20	30	30	27	30
SMG009U	19	20	29	32	32	20	27	25	31	27	32	31
SMG010F1	**17**	19	25	31	31	22	**6**	**10**	22	**12**	24	25
SMG010U	19	20	24	30	30	19	**6**	**11**	22	**14**	21	30
SMG011F7	18	19	30	32	33	21	28	22	30	23	25	32
SMG011U	24	26	33	>	37	23	34	24	>	30	>	36
SMG012F1	19	19	30	30	32	24	**13**	25	30	29	30	32
SMG012F3	**17**	19	28	29	31	21	26	19	25	24	24	26
SMG012F6	18	20	27	26	32	22	23	22	30	27	28	30
SMG012U	25	27	35	>	41	22	32	26	>	33	>	39
SMG013F1	**16**	18	28	28	33	21	**6**	19	29	27	26	31
SMG013U	20	21	28	33	34	20	22	25	29	27	31	31

Table S1 continued—Antimicrobial susceptibility of strains by disc-diffusion.

Strains	Antibiotics (mm)											
	**AMK**	**GEN**	**IPM**	**MEM**	**ETP**	**TGC**	**CIP**	**FOX**	**ATM**	**CAZ**	**CTX**	**FEP**
SMG014F10	**10**	**9**	31	33	32	22	**6**	21	**16**	**18**	**10**	**17**
SMG014F11	**10**	**8**	32	32	34	22	**6**	**18**	26	28	29	32
SMG014F12	**8**	**6**	32	34	30	22	**6**	21	**8**	**6**	**16**	**14**
SMG014U	19	**12**	31	30	30	23	**6**	22	**13**	**14**	**10**	**15**
SMG015U	21	19	29	32	32	19	31	26	32	27	31	32
SMG016F1	19	20	31	32	34	25	32	22	32	30	32	34
SMG016F4a	20	22	28	30	33	25	30	24	27	26	33	29
SMG016F10	18	18	29	36	31	23	30	23	26	27	27	30
SMG016U	23	25	31	35	36	25	34	28	>	>	>	35
SMG017F1	**17**	19	29	31	30	21	27	23	27	25	24	26
SMG017F2	**17**	18	27	30	31	20	28	25	26	25	24	26
SMG017F3	**17**	19	26	29	31	20	30	24	25	23	23	25
SMG017F8	18	19	28	32	33	21	26	21	28	27	27	31
SMG017U	20	21	30	33	33	21	30	25	>	27	33	31
SMG018F1	19	21	30	32	33	18	31	19	28	25	27	30
SMG018U	20	17	34	34	33	22	28	23	31	26	34	31

Values of the diameters in millimeters. AMK: amikacin; GEN: gentamicin; IPM: imipenem; MER: meropenem; ETP: ertapenem; TGC: tigecycline; CIP: ciprofloxacin; FOX: cefoxitin; ATM: aztreonam; CAZ: ceftazidime; CTX: cefotaxime; FEP: cefepime. The antimicrobial susceptibility was determined according to EUCAST and represented by the text colors; blue values mean susceptible, standard dosing regimen; orange values mean susceptible, increased exposure; bold red values mean resistance. The symbol > represents a diameter of more than 40 mm.

Table S2—Antimicrobial resistance genes, point mutations, and ST of the 49 *Escherichia coli* strains sequenced.

Strains	ST (cc)	AMR genes detected [a]	punctual mutations
SMG001F1	38	*aph(3'')-Ib, aph(6')-Id,* $bla_{\text{TEM-30}}$, *catA1, dfrA7, sul2, tetD*	*gyrA* p.S83L
SMG001F6	38	*aph(3'')-Ib, aph(6')-Id,* $bla_{\text{TEM-30}}$, *catA1, dfrA7, sul2, tetD*	*gyrA* p.S83L
SMG001F2	6458 (10)	*aadA2, aph(3'')-Ib, aph(6')-Id,* $bla_{\text{TEM-1C}}$, *dfrA12, sul1, tet(A)*	-
SMG002F4	10	-	-
SMG002F6	10	-	-
SMG002F9	10	-	-
SMG002F5	88	*aadA1, dfrA1, sul2, tet(B)*	-
SMG002F8	1727 (446)	-	-
SMG003F2	69	*aph(3'')-Ib, aph(6')-Id, sul2*	*gyrA* p.S83L
SMG003F3	69	*aph(3'')-Ib, aph(6')-Id, sul2*	*gyrA* p.S83L
SMG003F7	13	*aadA1, dfrA1, floR, sul2*	-
SMG003F10	13	*aadA1, dfrA1, floR, sul2*	-
SMG004F1	69	$bla_{\text{TEM-1B}}$	*gyrA* p.S83L
SMG004F8	69	$bla_{\text{TEM-1B}}$	*gyrA* p.S83L
SMG005F2	1193 (14)	-	*gyrA* p.S83L<br>*gyrA* p.D87N
SMG005F3	1193 (14)	-	*gyrA* p.S83L<br>*gyrA* p.D87N
SMG006F1	10	-	-
SMG006F11	1193 (14)	*aac(3)-IId,* $bla_{\text{TEM-1B}}$	*gyrA* p.S83L<br>*gyrA* p.D87N
SMG007F1	14	-	-
SMG007F7	14	-	-
SMG007U	14	-	-
SMG008F1	101	-	-
SMG008F10	101	-	-
SMG008F2	10	*aac(6')-Ib-cr, aadA1, aph(3')-Ia, aph(3'')-Ib, aph(6')-Id,* $bla_{\text{OXA-1}}$, $bla_{\text{CTX-M-15}}$, $bla_{\text{TEM-1B}}$, *catB3, cml, dfrA14, floR, sul3, tet(B)*	*gyrA* p.S83L<br>*gyrA* p.D87N
SMG008F9	10	*aac(6')-Ib-cr, aadA1, aph(3')-Ia, aph(3'')-Ib, aph(6')-Id,* $bla_{\text{OXA-1}}$, $bla_{\text{CTX-M-15}}$, $bla_{\text{TEM-1B}}$, *catB3, cml, dfrA14, floR, sul3,* ***tet(A)***, *tet(B)*	*gyrA* p.S83L<br>*gyrA* p.D87N
SMG008U	10	*aac(6')-Ib-cr, aadA1, aph(3')-Ia, aph(3'')-Ib, aph(6')-Id,* $bla_{\text{OXA-1}}$, $bla_{\text{CTX-M-15}}$, $bla_{\text{TEM-1B}}$, *catB3, cml, dfrA14, floR, sul3,* ***tet(A)***, *tet(B)*	*gyrA* p.S83L<br>*gyrA* p.D87N
SMG008F11	69	*aadA5, aph(3'')-Ib, aph(6')-Id,* $bla_{\text{TEM-1B}}$, *dfrA17, sul2*	-
SMG009F1	73	-	-
SMG009F5	219	-	-
SMG010F1	224	$bla_{\text{TEM-1B}}$, $bla_{\text{DHA-1}}$, *qnrB4, su1l, tet(B),*	*gyrA* p.S83L<br>*gyrA* p.D87N
SMG011F7	5493 (20)	-	-

Table S2 Continued—Antimicrobial resistance genes, point mutations, and ST of the 49 *Escherichia coli* strains sequenced.

SMG012F1	101	*aac(3)-IId, aph(3′)-Ia, aph(3′′)-Ib, aph(6′)-Id, aadA1, aadA2,* $bla_{\text{TEM-1B}}$, *dfrA12, floR, sul2, tet(B)*	*gyrA* p.S83L<br>*gyrA* p.D87N
SMG012F3	361	-	-
SMG012F6	69	*mcr-1.26, qnrB19*	-
SMG013F1	131	-	*gyrA* p.S83L<br>*gyrA* p.D87N
SMG013U	998	*qnrB19*	-
SMG014F10	131	*aac(3)-IIa, aac(6′)-Ib-cr, aph(3′′)-Ib, aph(6′)-Id,* $bla_{\text{OXA-1}}$, $bla_{\text{CTX-M-15}}$, $bla_{\text{TEM-1B}}$, *catA1, catB3, sul2, tet(B)*	*gyrA* p.S83L<br>*gyrA* p.D87N
SMG014F12	131	*aac(3)-IIa, aac(6′)-Ib-cr, aph(3′′)-Ib, aph(6′)-Id,* $bla_{\text{OXA-1}}$, $bla_{\text{CTX-M-15}}$, $bla_{\text{TEM-1B}}$, *catA1, catB3,* ***dfrA14, qnrB1****, sul2,* ***tet(A),*** *tet(B)*	*gyrA* p.S83L<br>*gyrA* p.D87N
SMG014F11	1193 (14)	*aac(3)-IId, aadA5,* $bla_{\text{TEM-1B}}$, *dfrA17, sul1*	*gyrA* p.S83L<br>*gyrA* p.D87N
SMG015U	1236	-	-
SMG016F1	69	*aph(3′′)-Ib, aph(6′)-Id, sul2*	-
SMG016F4a	69	-	-
SMG016F10	69	-	-
SMG017F1	69	*tet(B)*	-
SMG017F2	93	-	-
SMG017F3	59	-	-
SMG017F8	4238 (10)	-	-
SMG017U	127	*aph(3′′)-Ib, aph(6′)-Id,* $bla_{\text{TEM-1B}}$, *sul2*	-
SMG018F1	131	*aph(3′′)-Ib, aph(6′)-Id,* $bla_{\text{TEM-1B}}$, *dfrA8, sul2, tet(A)*	-

[a] AMR genes described are those found using ResFinder in each strain. [b] In bold are the genes that were not found in the genome of the putative variants sequenced.
